# The synthesis of extracellular vesicles by the protistan parasite *Blastocystis*

**DOI:** 10.3389/fcimb.2022.1019789

**Published:** 2022-10-27

**Authors:** Steven Santino Leonardi, Eileen Yiling Koh, Lei Deng, Chenyuan Huang, Lingjun Tong, Jiong-Wei Wang, Kevin Shyong-Wei Tan

**Affiliations:** ^1^ Laboratory of Molecular and Cellular Parasitology, Healthy Longevity Translational Research Programme and Department of Microbiology and Immunology, Yong Loo Lin School of Medicine, National University of Singapore, Singapore, Singapore; ^2^ Department of Surgery, Yong Loo Lin School of Medicine, National University of Singapore, Singapore, Singapore; ^3^ Nanomedicine Translational Research Programme, Centre for NanoMedicine, Yong Loo Lin School of Medicine, National University of Singapore, Singapore, Singapore; ^4^ Cardiovascular Research Institute, National University Heart Centre Singapore, Singapore, Singapore; ^5^ Department of Physiology, Yong Loo Lin School of Medicine, National University of Singapore, Singapore, Singapore

**Keywords:** *Blastocystis*, gut microbiome, extracellular vesicles (EVs), cytokines, ultracentrifugation

## Abstract

*Blastocystis* is a genus of single-celled protist belonging to the stramenopile group. Prior studies have shown that isolates of *Blastocystis* subtype 7 (ST7) induced higher levels of intestinal epithelial cell damage and gut microbiota dysbiosis in comparison to other subtypes in *in vivo* and *in vitro* settings. Prior research has shown a link between gut dysbiosis and exposure to extracellular vesicles (EVs) produced by pathogenic microorganisms. This study demonstrates a protocol for the isolation of EVs from *Blastocystis* ST7 *via* ultracentrifugation. Nanoparticle tracking analysis and transmission electron microscopy were used to assess EV size and morphology. The protein content of isolated EVs was assessed by mass spectrophotometry and the presence of EV markers were evaluated by Western blotting. Finally, the EVs were cocultured with prominent human gut microbiome species to observe their effect on prokaryote growth. Our data shows that *Blastocystis* ST7 secretes EVs that are similar in morphology to previously characterized EVs from other organisms and that these EVs contain a limited yet unique protein cargo with functions in host-parasite intercellular communication and cell viability. This cargo may be involved in mediating the effects of *Blastocystis* on its surrounding environment.

## Introduction

*Blastocystis* is a genus of unicellular stramenopilic gut parasites (Silberman et al., 1996), classified into over 20 subtypes (STs) (Stensvold et al., 2007). Human and animal infection by this parasite is ubiquitous, however it is limited by region and host species on a per-ST basis (Parkar et al., 2010; Alfellani et al., 2013). Infection occurs *via* the fecal-oral route, and is far more prevalent in developing nations (Tan, 2008). Variations in virulence have been observed both between and within *Blastocystis* subtypes (Wu et al., 2014b; Ajjampur and Tan, 2016). This paper focuses on ST7, isolates -B and -H. This subtype, in particular these isolates, have been observed to exhibit more virulent characteristics in comparison to the other commonly-studied human-infecting subtypes ST1 and ST4 (Yason et al., 2016; Yason et al., 2019).

The process of *Blastocystis* colonization remains unclear, though research has identified legumain and cathepsin B-like proteases on the surface of the *Blastocystis* cell as critical virulence factors (Wu et al., 2010; Wawrzyniak et al., 2012; Nourrisson et al., 2016). The process by which the parasite attaches to intestinal epithelial cells is of particular importance, as it is immotile and cannot actively travel to the site of infection (Boorom et al., 2008). *Blastocystis* infection has been associated with both positive and negative health outcomes in the host. It has been correlated with increases in the biodiversity of the microbiome (Scanlan et al., 2014; Audebert et al., 2016) as well as inflammatory disorders such as irritable bowel syndrome (IBS) (Kesuma et al., 2019) and ulcerative colitis (Stensvold et al., 2013), cell damage-induced disorders such as colorectal cancer (Kumarasamy et al., 2017; Sulżyc-Bielicka et al., 2021), and dysbiosis of the gut microbiome (Beghini et al., 2017; Tito et al., 2018; Billy et al., 2021). Koch’s postulates remain unproven, however, and a definitive causal link between the parasite and a disease has yet to be found.

‘Extracellular vesicles’ (EVs) is a collective term for three types of biogenesis-dependent vesicle that are secreted by living cells (Shao et al., 2018; Huang et al., 2021). Bacteria, mammalian cells, and unicellular parasites have been shown to secrete EVs implicated in cellular communication, regulation, and virulence pathways (Twu et al., 2013; Olmos-Ortiz et al., 2017; Liu et al., 2018; Reclusa et al., 2020). EVs are understood to contain species-dependent proteins, lipids, and nucleic acids, however their function and generation is poorly understood (Twu et al., 2013; Artuyants et al., 2020; Elzanowska et al., 2020). EVs produced by both pathogenic and commensal microbiota species have been implicated in the alteration of gut homeostasis (van Bergenhenegouwen et al., 2014; Chang et al., 2020; Cuesta et al., 2021). In particular, both *Entamoeba* and *Giardia* EVs were shown to activate host immune and cytokine responses in an *in vitro* model (Evans-Osses et al., 2017; Sharma et al., 2020).

Host-parasite interactions are commonly considered in gross molecular terms, while information exchange between the host and the parasite is poorly understood. The study of EVs is an intriguing putative avenue for this information exchange. Understanding of *Blastocystis*-produced EVs remains in a nascent stage, despite first being reported in 2001 (Tan et al., 2001). In this study, we report that *Blastocystis* subtypes 7B and 7H secrete EVs with similar physical characteristics and protein components to other microorganismal systems, and show that these EVs are capable of influencing *in vitro* host cells and microbiota species.

## Results

### *Blastocystis* ST7 secretes extracellular vesicles

**Figure 1:**
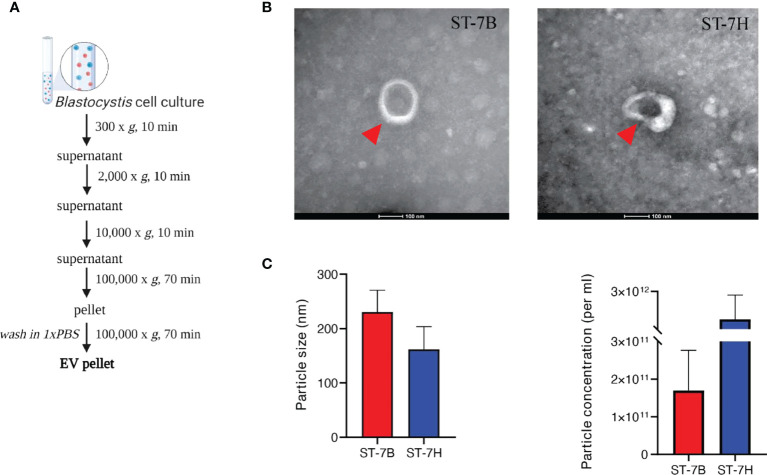
Characterization of extracellular vesicles from *Blastocystis* ST7 cultured in growth medium. **(A)** EV isolation protocol, adapted from Théry et al. (Théry et al., 2006). **(B)** Transmission electron microscopy of EVs from ST7B and ST7H. One EV is depicted in each image, indicated by the red arrow. Scale bar = 100nm. **(C)** Mean EV size and concentration produced by ST7B and ST7H under identical culture conditions.

To determine whether *Blastocystis* ST7 secretes EVs, axenic isolates were maintained in IMDM supplemented with 5% horse serum at 37°C in anaerobic conditions. Conditioned medium was collected and subjected to a series of centrifugation cycles adapted from Théry et al. (Théry et al., 2006) (**Figure 1A**). The recommendations of the International Society for Extracellular Vesicles (ISEV) were used as a framework for vesicle characterization (Thery et al., 2018). Visualization using transmission electron microscopy (TEM) showed cup-shaped morphology typical to EVs (Rikkert et al., 2019) (**Figure 1B**). *Blastocystis* ST7B secreted an average of 1.70*10^11^ particles mL^-1^, while ST7H averaged 1.05*10^12^. Using nanoparticle tracking analysis (NTA), average EV size was measured at 230.4 ± 69.5 mm and 161.6 ± 72.9 mm for 7B and 7H respectively (**Figure 1C**). This data demonstrates that the collected media contained vesicles with a size and shape similar to those produced by other protists (Twu et al., 2013; Sharma et al., 2020).

### *Blastocystis* ST7 EV proteins cover biological GO terms uncommon in the parasite

**Figure 2:**
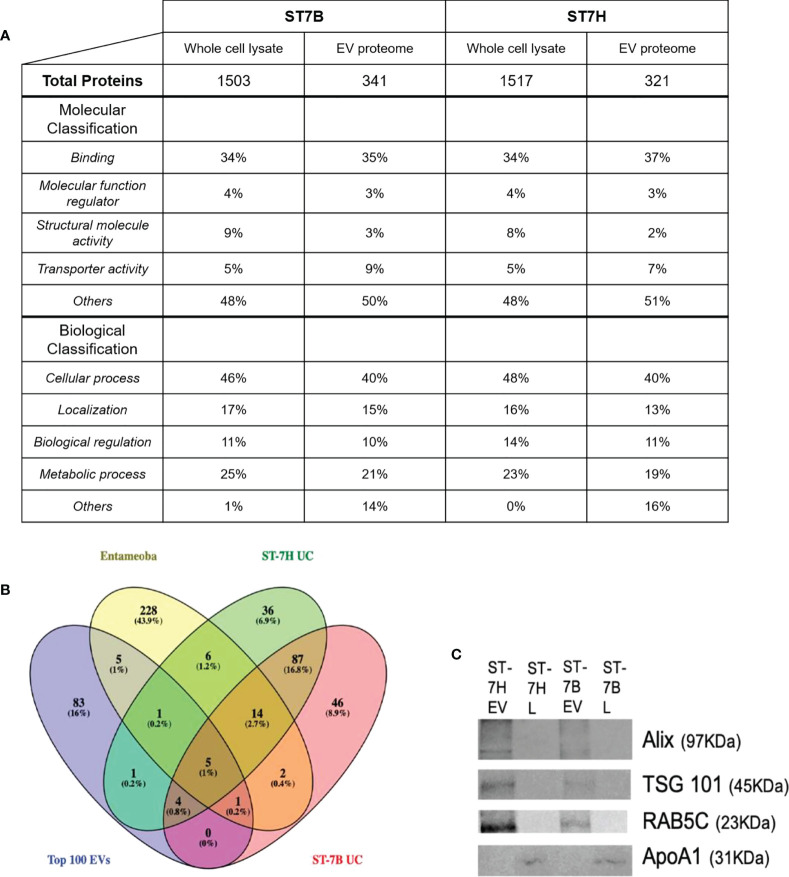
Functional analysis of *Blastocystis* ST7 EV proteome. **(A)** Table comparing proteins present in ST7 EVs and whole cell lysate. Proteins were identified using mass spectrophotometry. Protein function predicted using gene ontology analysis *via* the PANTHER database. **(B)** Venn diagram showing overlap between ST7B/ST7H EV proteins identified in this study, *Entamoeba histolytica* EVs identified by Sharma et al., 2020, and the top 100 EV markers as curated by the Vesiclepedia database. **(C)** Western blot analysis of endosomal sorting (RAB5C; 23 KDa), tumour suppressor gene (TSG) 101 (TSG101; 45KDa) and enrichment of vesicle trafficking (PDCD6IP/ALIX; 97 KDa) markers in isolated *Blastocystis* ST7 EVs and lysates (L), with apolipoprotein ApoA1 (31 KDa) serving as negative marker control (Thery et al., 2018). Each lane was loaded with 200 µg EV protein. Western blot shown here is representative of three runs.

Mass spectrophotometry was used to determine the protein content of ST7B and ST7H EVs and whole cell lysates (WCLs). Proteins with two or more peptides aligned to the UniProt database were included for downstream analysis. A total of 341 and 321 proteins were identified from ST7B and ST7H EV, along with 1503 and 1517 proteins from ST7B and ST7H WCLs respectively. Gene ontology (GO) analysis using the PANTHER database (Mi et al., 2021) showed a reduction in proteins associated with the molecular GO term ‘structural molecule activity’ in both assessed strains of ST7 EV relative to WCLs (**Figure 2A**). There was also a relative increase in proteins with transporter activity in ST7 EVs relative to WCLs. In both strains of WCL, the top four biological classification GO terms covered the vast majority (≥99%) of detected proteins. In EVs, the proportion of proteins comprising other biological GO terms outside of these four increased to 14 and 16% of the total.

### *Blastocystis* ST7 EVs contain proteins uncommon to EVs as a whole

The ST7 EV proteomes were filtered. Identified proteins with a coverage of >2% were excluded. The filtered EV proteomes were then compared with the *Entamoeba histolytica* EV proteome and the Top 100 most common EV proteins as listed on the Vesiclepedia database at time of analysis (Kalra et al., 2012; Sharma et al., 2020). Nine proteins were found to be shared between ST7 EVs and the Top 100 out of a total of 154 for ST7H and 159 for ST7B. This represents ~6% of the total number of observed proteins in each subtype EV. Nineteen proteins were shared between ST7 and *Entamoeba* EVs. ST7H EVs expressed more proteins in common with *Entamoeba* than ST7B. A significant portion of the assessed proteins, 46 (28.9% of 7B total) and 36 (23.4% of 7H total) were unique to that specific strain of *Blastocystis* ST7.

### *Blastocystis* ST7 EVs do not express the tetraspanin family of proteins

The tetraspanin family of proteins, known pan-EV markers (Yáñez-Mó et al., 2015) were absent from the ST7 EV proteome. This absence has also been observed in other parasite-derived EVs (Tzelos et al., 2016; Arab et al., 2019; Hansen et al., 2019). NCBI BLAST validation against an existing *Blastocystis* genome reference (Denoeud et al., 2011) identified no homologs to the tetraspanin family within the genetic code.

### Observed *Blastocystis* ST7 EV protein data are not due to WCL contamination

Western blotting was employed to confirm a lack of WCL contamination in existing EV samples (**Figure 2C**). The proteins ALIX (programmed cell death 6 interacting protein), TSG101 (tumour susceptibility gene 101), and RAB5C (a member of the RAS oncogene family), which were identified in our mass spectrophotometry were likewise present in our western blotting. APOA1 (apolipoprotein A1) was used as an EV purity control, as it has been previously in the literature as an indicator of WCL contamination (Karimi et al., 2018; Thery et al., 2018). APOA1 was not observed in our EV samples.

### *Blastocystis* ST7 EVs induce death in HT29 cells

**Figure 3:**
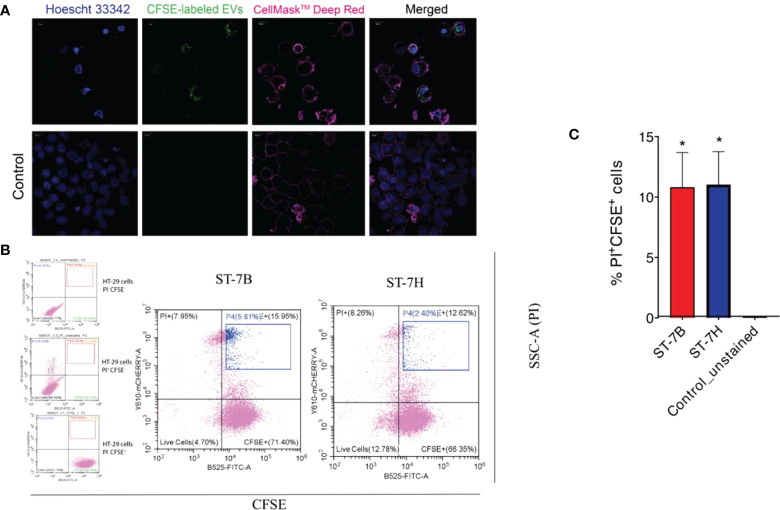
Uptake of *Blastocystis* EVs by HT29 cells leads to an increase in cell death. **(A)** Confocal microscopy images showing HT29 cells taking up *Blastocystis* EVs (HT29 nuclei stained blue with Hoescht 33342, HT29 cell membrane stained pink with CellMask™ Deep Red, EVs stained green with CFSE). Scale bars = 10 µm. **(B)** Flow cytometry analysis of HT29 uptake of *Blastocystis* EVs. FITC signal (x-axis) represents CFSE staining; mCherry signal (y-axis) represents propidium iodide staining. Cells in the blue gate have taken up EVs and are nonviable. **(C)** Proportion of nonviable cells with CFSE-stained EV uptake presented as mean ± SEM. n=3 independent experiments. *p<0.05.

ST7 EVs were labeled using CFSE. HT29 cells were then incubated in the presence of varying concentrations of labelled EVs for one hour at 37°C. Confocal imaging showed uptake and internalization of the labelled EVs at all concentrations (**Figure 3A**). Some cells showed visible signs of viability loss, particularly membrane blebbing. The cells were stained with propidium iodide (PI) and assessed *via* flow cytometry (**Figure 3B**). HT29 cells incubated with ST7 EVs showed increased PI fluorescence. 10.8% and 11% of cells were positive for PI when incubated with ST7B and ST7H respectively, in comparison with <1% of control cells (**Figure 3C**).

### *Blastocystis* ST7 EVs can influence *B. longum* and *E. coli* growth

In a 2019 study, our lab showed that coculture with *Blastocystis* ST7 can influence the growth of some gut bacteria species (Yason et al., 2019). Here, we investigated whether similar effects are observed during coculture with ST7 EVs. We cocultured *E. coli, B. longum*, and *L. brevis* alternately with a standard number of EVs (10^7^), as well as the concentration of EVs required to observe the effect in the previous experiment (80 µg). *B. longum* growth was reduced when cultured with ST7 EVs, while *E. coli* growth increased when cultured with ST7B EVs. *L. brevis* remained unaffected. These results are similar to those observed in the above study.

### *Blastocystis* ST7B EVs can influence THP1 cell inflammatory cytokines

A 2014 study by Teo et al. (Teo et al., 2014) showed that *Blastocystis* ST7, including isolate ST7B, is capable of altering NF-κB levels in THP1-Blue cells. We used qPCR to assess whether ST7B EVs can induce changes in three NF-κB-associated cytokines: IL-1β, IL-6, and TNF-α. When cocultured with ST7B EVs, IL-1β expression significantly increased, while IL-6 expression decreased.

## Discussion

This study is the first to identify and characterize the EVs synthesized by *Blastocystis*, drawing attention to their putative role in parasite-host interactions. The two ST7 isolates (ST7B and ST7H) used in this study have been reported to be more virulent (Wu et al., 2014a; Wu et al., 2014b), display increased resistance to antiparasitic drugs (Mirza et al., 2011), and induce host immune responses to a greater extent (Yason et al., 2016) compared to other *Blastocystis* subtypes. In particular, *Blastocystis* ST4 has been shown to have a beneficial effect on the host (Deng et al., 2021; Deng and Tan, 2022). Currently, the divergence in observed pathogenicity of these and other subtypes has not been explained, and differences in EV characteristics offer a potential solution.

We showed that ST7 EVs conform to typical EV size and structure as characterized elsewhere (Twu et al., 2013; Evans-Osses et al., 2017). We also established the number of EVs synthesized by a consistent number of *Blastocystis*; these will be useful points of comparison in future studies (**Figure 1**). We defined the ST7 EV proteome *via* mass spectrophotometry and used western blotting to confirm the presence of canonical EV proteins ALIX, TSG101, and RAB5C (**Figure 2**). This confirms that we were able to successfully isolate EVs originating from *Blastocystis*. The absence of the pan-EV marker tetraspanin was noted, however this may be another instance of the absence of tetraspanins from parasite-produced EVs (Tzelos et al., 2016; Arab et al., 2019; Hansen et al., 2019).

Gene Ontology analysis of the identified EV proteins showed an enrichment of biological classification GO terms not common in the WCL. Proteins not described by the top four biological GO terms comprised approximately less than 1% of the ST7 WCL, compared to ~15% of the ST7 EV proteome. Similarly, 169 of the 203 filtered ST7 EV proteins assessed against Vesiclepedia in **Figure 2B** were found to be unique to *Blastocystis*. A further 82 of those proteins were unique to either ST7B or ST7H. This suggests that *Blastocystis* EVs can possess a unique protein cargo that diverges even between isolates of a single subtype. EVs in general have been noted to exhibit significant variability, so further research must be performed to confirm these results (Tiruvayipati et al., 2020; Newman et al., 2021).

**Figure 4:**
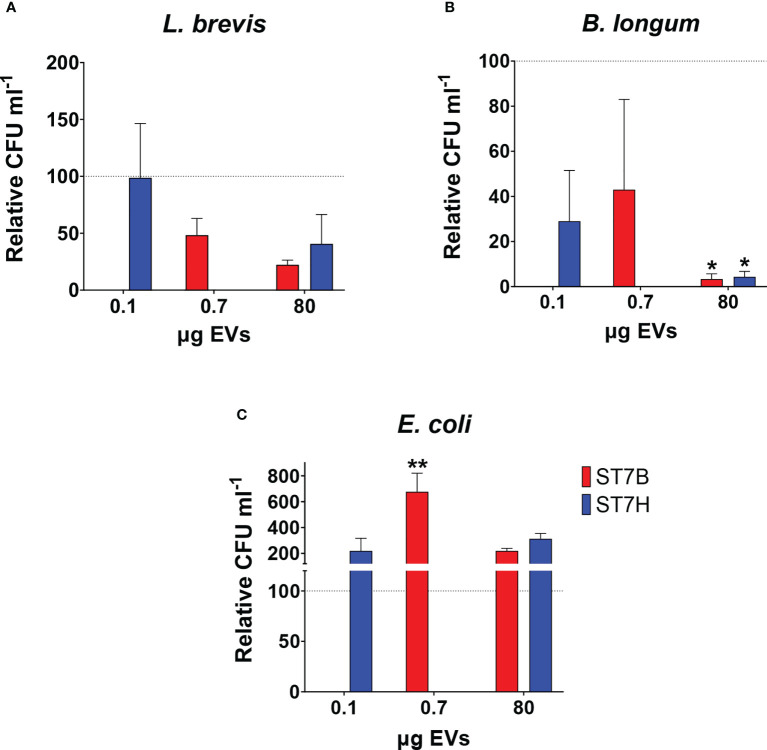
*Blastocystis* ST7 EVs cause a reduction in *B longum* while increasing *E coli* growth. Coculture of *Blastocystis* ST7 EVs with three species of physiologically relevant human gut microbiota in PBS: **(A)** *Lactobacillus brevis*, **(B)** *Bifidobacterium longum*, and **(C)** *Escherichia coli*. Bars represent level of prokaryote growth following 24 hours coculture as mean ± SD. Two standard quantities of EV were used: 80 µg, the amount required to produce the effect observed in **Figure 3B**, and 10^7^ EVs (converted to µg in this figure). Statistical testing performed using two-way ANOVA; n=3. Statistical significance asterisks show significance relative to control organisms not supplemented by EVs (shown as the dotted horizontal line). **p<0.05.

**Figure 5:**
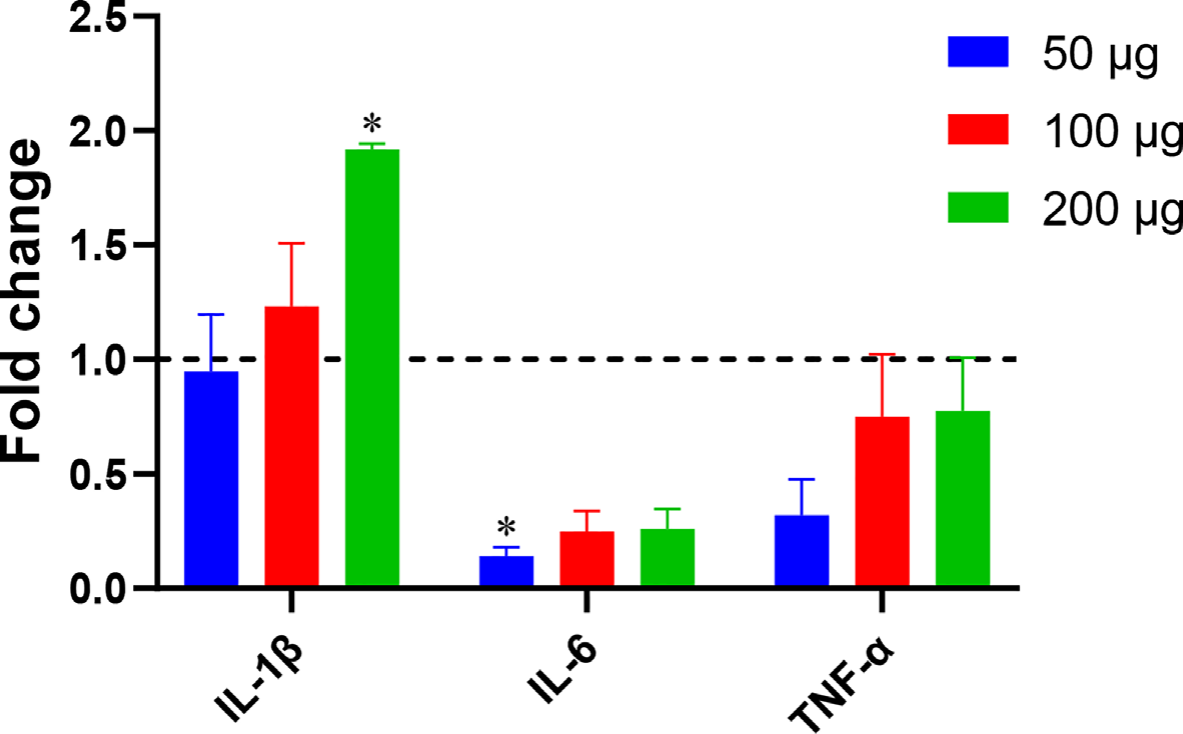
*Blastocystis* ST7B EVs alter inflammatory marker levels in a human immune cell model. THP1 cells were incubated with varying concentrations of ST7B EVs. qPCR was performed to assess concentrations of three inflammation-promoting proteins: IL-1β, IL-6, and TNF-α. Bars represent DNA expression fold change relative to a control cultured without EVs. Statistical testing performed using two-way ANOVA; n=3. Statistical significance asterisks show significance relative to cells not supplemented with EVs (shown as dotted horizontal line).

**Figure 6:**
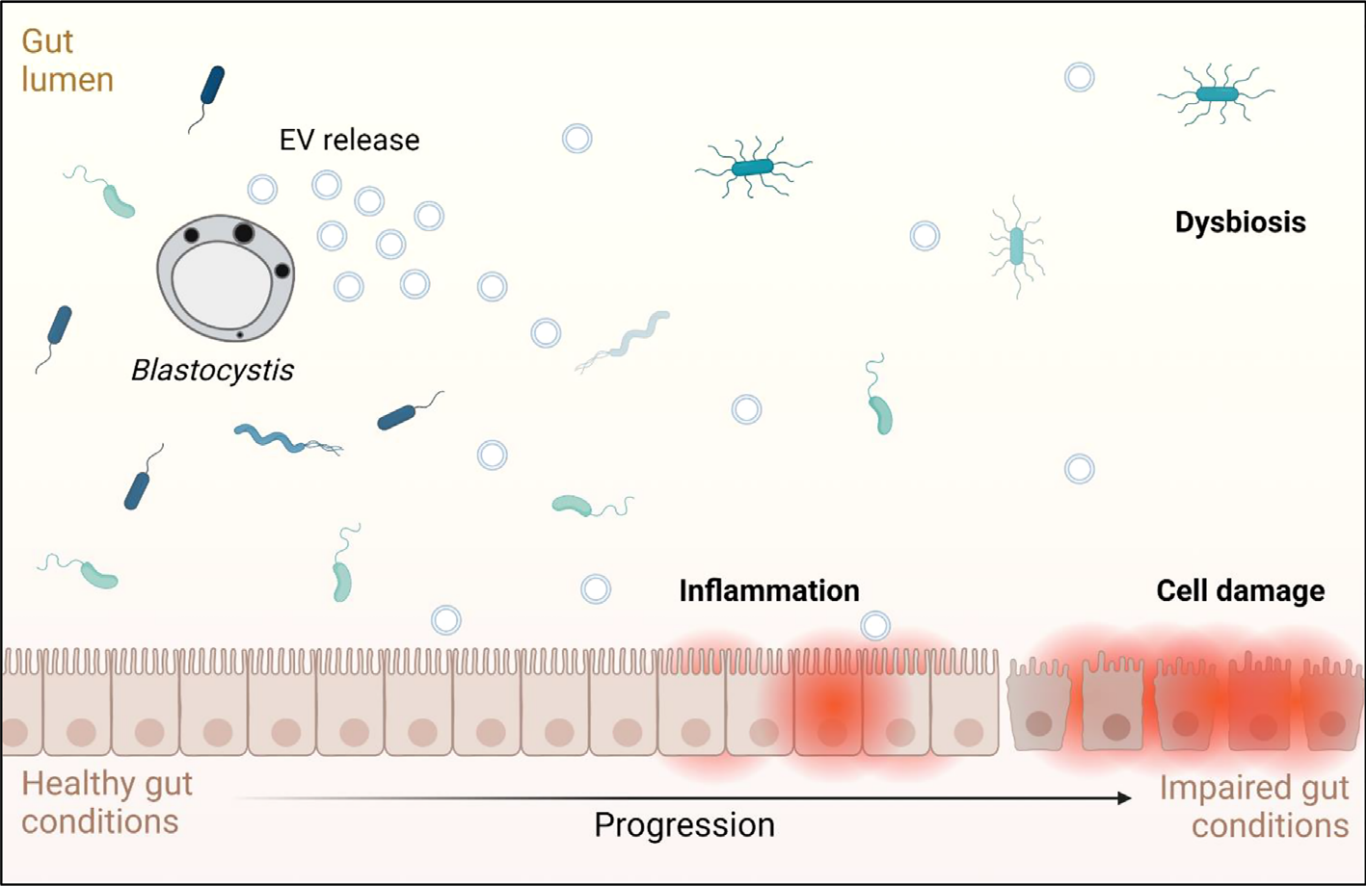
Graphical representation of the effects *Blastocystis* EVs may have on a human host, based on the results observed in this study and on conditions associated with *Blastocystis* infection. Adapted from “Impaired Gut Membrane”, by BioRender.com (2022). Retrieved from https://app.biorender.com/biorender-templates.

Fluorescence microscopy demonstrated that ST7 EVs can be taken up by a human *in vitro* model cell line, and that they have a negative effect on the viability of those cells as represented by an increase in propidium iodide uptake (**Figure 3**). We demonstrated effects of ST7 EVs on gut microbiota (**Figure 4**) consistent with those previously observed during *Blastocystis* – prokaryote coculture experiments (Yason et al., 2019) – specifically, an inhibition of the beneficial gut species *B. longum* (Wong et al., 2019) when incubated with a high concentration of EVs. Finally, we characterized the influence of ST7B EVs on inflammatory cytokines within differentiated THP-1 cells, showing that IL-1β expression increases with an increasing concentration of ST7B EVs (**Figure 5**). IL-1β is a pro-inflammatory cytokine associated with gastrointestinal cancer and T-cell activation (Lu et al., 2005; Ben-Sasson et al., 2013). The effects of ST7 EVs on *E. coli* growth and IL-6 expression were statistically significant but not dose-dependent, necessitating further research to validate the observed effects. Our results show evidence for *Blastocystis* EVs inhibiting the growth of a beneficial gut prokaryote, reducing the viability of a human gastrointestinal cell model, and increasing the expression of a pro-inflammatory, cancer-inducing cytokine in that same model. These correlate with dysbiosis, inflammation, and damage to the host gut epithelium – all features of diseases associated with *Blastocystis* (**Figure 6**). This suggests that *Blastocystis* EVs are likely to play a role in the deleterious effects the parasite has been observed to induce in the gut.

## Conclusion

The avenues by which beneficial and pathogenic *Blastocystis* subtypes affect host cells and host microbiota remain poorly characterized. Understanding these avenues will help shed light on the source of *Blastocystis* virulence and the reasons for highly divergent behaviour between *Blastocystis* subtypes. This paper presents EVs as a likely mediator of interactions between *Blastocystis*, the microbiome, and the host. Future work can use our experiments as embarkation points to investigate the results shown here in greater depth, with an eye on establishing a causal relationship between EVs and effects, or investigating more complex model organisms. Of importance is the need to determine whether these effects are observable in other *Blastocystis* subtypes, and if so, whether their degree of severity correlates with the virulence of the subtype and isolate.

## Materials & methods

### Blastocystis culture

Human *Blastocystis* isolates were acquired from patients at the Singapore General Hospital in the early 1990s, before the establishment of the Institutional Review Board at the National University of Singapore (NUS). Both the *Blastocystis* ST7 isolates -B and -H are maintained at the microbial collection at the Department of Microbiology and Immunology, NUS. Previously axenized cultures of both *Blastocystis* ST7B and ST7H (Ho et al., 1993) were maintained in pre-reduced Iscove’s Modified Dulbecco’s Medium (IMDM) (Gibco, New Zealand) supplemented with heat-inactivated 5% horse serum (Gibco, New Zealand) These cultures were incubated anaerobically with Anaerogen gas packs (Oxoid, USA) at 37°C in jars and subcultured on a weekly basis.

### HT-29 cell culture

Human colorectal adenocarcinoma cell line HT-29 (ATCC Cat. No. HTB-38) cells were maintained in T-75 flasks (Corning, USA) in a humidified incubator with 5% CO2 at 37°C. The Dulbecco’s Modified Eagle’s Medium (DMEM; Thermo Scientific, USA) was supplemented with 10% heat-inactivated FBS (Gibco, USA) and 1% each of penicillin-streptomycin, non-essential amino acids (Gibco, USA) and sodium pyruvate (Gibco, USA), and designated as complete medium.

HT-29 cells were seeded at approximately 1 x 10^5^ cells mL^-1^ per well and incubated for 48 hours to achieve at least 80% confluency, followed by synchronization of cells with serum-free DMEM for 24 hours. The HT-29 cells were then used for confocal imaging supplemented with EVs at 80 µg and/or ~1 x 10^7^ *Blastocystis* ST7 (whole) cells mL^-1^, respectively. HT-29 cells in culture medium without EVs were included as control.

### Bacterial cultures

*Escherichia coli* (ATCC 11775), *Bifidobacterium longum* (ATCC 15707) and *Lactobacillus brevis* (ATCC 14869) were cultured and maintained on Luria-Bertani (LB), Bifidus Selective Medium (BSM) and deMan, Rogosa, Sharpe (MRS) medium (all from Sigma-Aldrich, USA), respectively, in broth and agar formats. All cultures were incubated in an anaerobic jar with AnaeroGen™ gas pack (Oxoid, USA) at 37°C. Absorbance readings of bacterial broth cultures prior to experiments were done using the spectrophotometer at 600 nm wavelength.

### Enrichment of EVs by differential ultracentrifugation

An ultracentrifugation protocol established by Théry et al. (Thery et al., 2018) was used to isolate EVs, where serum-free cell culture medium (CCM) was centrifuged at 300 x g for 10 minutes at 4°C to remove larger cells by pelleting. The supernatant was transferred to a new tube and centrifuged at 2000 x g for 10 minutes at 4°C to pellet dead cells, followed by another round of centrifugation at 10,000 x g for 30 minutes at 4°C to collect the cell debris. Clarified CCM was then centrifuged in an Optima™ L-90 ultracentrifuge (Beckman Coulter, USA) at 100,000 x g for 70 minutes at 4°C with a SW41Ti swinging-bucket rotor (Beckman Coulter, USA) to pellet EVs, which are washed with 1xPBS and re-centrifuged for another 70 minutes at 4°C at 100,000 x g. The resulting EV pellet was resuspended in 100μL of 1xPBS and stored at -80°C until usage.

### Transmission electron microscopy

Freshly isolated EVs were prepared for visualization by transmission electron microscopy (TEM) by fixation with 2.5% glutaraldehyde at 4°C for 1 hour. A volume of 20 µL per sample were incubated for 5 minutes on a Formvar Film 200 mesh, CU, FF200-Cu grid (Electron Microscopy Sciences, USA). Negative staining was then performed by incubating with 2.5% gadolinium triacetate for 1 minute. Fixed samples were viewed on the FEI TECNAI SPIRIT G2 (FEI Company, USA) at room temperature. Microscopy images were acquired from three independent experiments with three technical replicates (n=9).

### Nanoparticle tracking analysis

EVs were diluted in filtered phosphate-buffered saline to the optimal concentration for data acquisition. Five 60-second video clips were recorded at room temperature. These video clips were subsequently analyzed with detection threshold of 4-6 with NanoSight NTA software v3.2 (Nanosight, United Kingdom). Data were acquired from three independent experiments with three technical replicates (n=9).

### Assessment of total protein content by mass spectrophotometry

Proteins were extracted from the UC- isolated EVs by homogenization with lysis buffer (50mM triethylammonium bicarbonate, 8M urea, 1% sodium deoxycholate) at a 1:2 pellet volume to lysis buffer ratio. *Blastocystis* ST7B and ST7H cell lysates were included as controls. The mixture was incubated at room temperature for 20 minutes with vortex every 5 min, followed by centrifugation at ≥21,000 x g at 4°C for 30 minutes. Resultant supernatant was transferred to new microcentrifuge tube and analyzed. EVs and cell lysate protein samples were analyzed in two biological replicates over two independent technical runs. Trypsin digestion of samples were performed before using the TripleTOF^®^ 5600 System (Sciex, USA).

The protein sequences were searched against the UniProt protein database using ProteinPilotTM Software v5.0 (Sciex, USA). The Paragon search algorithm was used with the following parameters set to default values: trypsin specificity, cys alkylation, thorough false discovery rate (FDR). *Blastocystis hominis* was used as the reference organism (Denoeud et al., 2011). Only peptides occurrence of ≥ 2 at 95% confidence levels (CI) with FDR of 1% were taken into consideration. Venn diagram of *Blastocystis* ST7 EV proteins that were unique, shared and common to *Entameoba histolytica* EVs (Sharma et al., 2020) and the Top 100 commonly identified EV proteins (Kalra et al., 2012) were generated using the online version of Venny v2.1.0 (Oliveros, 2007). Functional pathways associated with EV proteins identified from *Blastocystis* ST7 were inferred by KEGG analysis (Kanehisa Laboratories, Japan), and Gene Ontology (GO) annotation results were obtained using the PANTHER database (Mi et al., 2021).

### Western blot

*Blastocystis*-derived EVs were lysed with RIPA (Invitrogen, USA) containing Protein Inihibitor (100x) on ice for 30 minutes. Proteins were quantified with the PierceTM BCA Protein Assay Kit (Thermo-Fisher Scientific, USA) on the Infinite^®^ 200 PRO NanoQuant (TECAN, USA). Approximately 200 µg of protein from each sample were lysed in 4x loading buffer (Invitrogen, USA) by heating for 10 minutes at 98°C; thereafter loading of the samples onto an SDS-PAGE gel for protein bands separation at 110 V for approximately 1 hour. The Spectra Multicolor Broad Range Protein Ladder (Thermo-Fisher, USA, Cat. No. 26623) was used for reference. Samples were transferred to nitrocellulose membranes and blocked with 5% BSA in Tris-buffered saline + 1% Tween20 (TBST), followed by overnight incubation with primary antibodies: rabbit monoclonal anti-ALIX (Abcam, USA, Cat. No. ab186429), rabbit monoclonal anti-RAB5C (Abcam, USA, Cat. No. ab199530), rabbit monoclonal anti-TSG101 (Abcam, USA, Cat. No. ab125011) and rabbit monoclonal anti-Apo-A1 (Abcam, USA, Cat. No. ab52945). Membranes were washed three times with TBST and mouse anti-rabbit HRP-conjugated secondary antibody incubation was carried out for 60 minutes at room temperature. Protein bands were visualized using the Electrogenerated Chemiluminescence (ECL) Western Blotting Substrate Kit (Millipore, USA, Cat. No. 1825002) and imaging was performed on a ChemiDoc (Bio-Rad Laboratories, Inc., USA). Data were acquired from three independent experiments.

### Cellular uptake of *Blastocystis*-derived EVs and live cell imaging by confocal microscopy

Purified *Blastocystis*-derived EVs were labeled with Vybrant^®^ CFDA SE Cell Tracer Kit (Invitrogen, USA; hereafter referred to as CFSE) as previously described (Morales-Kastresana et al., 2017). In brief, 100 µL of CFSE (200 µM) was added to 80 µg of EVs and the mixture were incubated for 15 minutes at 37°C. Cells were re-pelleted by centrifugation, resuspended in fresh pre-warmed PBS and incubated for a further 30 minutes to ensure complete modification of the probe. The CFSE-stained EVs were then washed once with PBS by centrifugation, followed by resuspension in serum-free culture medium.

For live confocal examinations of EV uptake and intracellular localization, 10^5^ cells mL^-1^ HT-29 cells were seeded into an 8-well µ-Slide (ibidi GmbH, Germany) and cultured as described above before the addition of CFSE-stained EVs at either 200 µg or 500 µg for co-incubation at 37 °C for 1 hour. The supernatant was subsequently replaced with a mixture consisting of both the Hoescht 33452 and CellMask™ Plasma Membrane Stain Deep Red (Thermo-Fisher, USA) dyes, with incubation at room temperature for 15 minutes before live imaging. All images were taken on the Olympus FV3000 Confocal Laser Scanning Microscope (Olympus Corporation, USA) with oil immersion (n=3), with at least three images taken per sample. Negative control consisting of only stained HT-29 cells without CFSE-stained EVs were included.

### Flow cytometry

The CFSE-stained EV uptake by HT-29 cells was quantified on a Beckman Coulter CytoFLEX Flow Cytometer (Beckman Coulter, USA). Cells were prepared as described for confocal microscopy, and were trypsinized with 0.25% EDTA-trypsin for 10 minutes at 37°C in the 5% CO2 incubator. The detached cells were pelleted by centrifugation followed by supernatant removal; a volume of 0.5mL of 1xPBS was used then to resuspend the cell pellet followed by the addition of 0.5 µL of PI (0.1mg mL^-1^) prior to flow cytometry. Data were calculated from three independent experiments with two technical replicates per sample (n=6).

### Co-culture of *Blastocystis*-derived EVs with bacterial cells

*L. brevis*, *B. longum* and *E. coli* cells were washed twice in PBS at 1000 x g for 10 minutes. Prior to the co-incubation, an aliquot of the cells was enumerated using the drop-plate method outlined in Yason et al. (Yason et al., 2019) to determine the number of initial cells. A concentration of 80 µg EVs were then incubated with 1mL of PBS-washed bacterial cells for 24h at 37°C in pre-reduced PBS under anaerobic conditions. After 24 hours, the bacterial numbers were counted and the bacterial colony-forming units (CFUs) mL^-1^ was determined when the colonies appeared on the agar plates. Controls containing only bacterial cells or co-cultured with 10^7^ *Blastocystis* cells mL^-1^ in pre-reduced PBS were included. The number of each bacterial cell type(s) were enumerated from three independent experiments with three technical replicates (n=9) and presented as % relative to control.

### THP1 cell culture

THP1 cell line (Invivogen, USA) were maintained in RPMI-1640 (Gibco, USA) supplemented with 10% heat-inactivated fetal bovine serum (FBS) (Gibco, USA), 100 U mL^-1^ penicillin, and 100 μg mL^-1^ streptomycin (ThermoFisher, USA). Cells were seeded onto a T-75 flask at a density of 2.5 × 106 cells in a humidified incubator at 37°C and 5% CO2. To differentiate THP1 cells into macrophages, cells were stimulated with 25 ng/mL phorbol 12-myristate 13-acetate (Sigma-Aldrich, USA) for 48 h. PMA-differentiated THP1 cells were then co-incubated with *Blastocystis* ST7-derived EVs (50, 100, and 200 ng mL^-1^, respectively) or PBS (negative controls) for 48 h.

### Gene expression analysis using RT-qPCR

Total RNA was extracted from *Blastocystis* ST7-derived EVs and PBS-treated THP1 cells using RNAzol RT (Sigma-Aldrich, USA) according to the manufacturer’s protocols. Complementary DNA was synthesized using the iScript cDNA kit (Bio-Rad, USA). The SsoAdvanced™ Universal SYBR Green Supermix (Bio-Rad, USA) was used in all qPCR amplifications on an Applied Biosystems 7500 Fast Real-Time PCR System (Applied Biosystems, USA). qPCR reaction was carried out in a total volume of 10 μL, which comprised of the master mixture and 2 μL of cDNA template. The former contained 5 μL SsoAdvanced™ Universal SYBR Green Supermix (2×), 0.3 mM of each primer, made up to 10 μL with nuclease-free water. Actin was used as a house-keeping gene, and three pro-inflammatory cytokines TNF-α, interleukin (IL)-6, and IL-1β were detected by qPCR. Fold change was determined by the 2−(ΔΔCt) method (Zhang et al., 2013).

### Statistical analysis

Data were presented as mean ± standard error of the mean (SEM) of triplicate experiments. Either two technical replicates (n=6) or three technical replicates (n=9), as described in the respective method sub-sections, were done. Acquired data were statistically analyzed using GraphPad Prism v8.0 (Swift, 1997). The analysis of variance (ANOVA; comparisons of more than two groups) were computed, with p values of <0.05 taken to be of statistical significance.

## Data availability statement

The original contributions presented in the study are publicly available. This data can be found here: https://data.mendeley.com/datasets/4r7xgpdrj4.

## Author contributions

KT and EK conceived the study and participated in its design and coordination. EK conducted experiments and organized collaborations with CH, LT, and J-WH pertaining to **Figures 1** and **2**. CH performed NTA analysis for **Figure 1C**, while LT performed protein work for **Figure 2C**. J-WH coordinated their work. DL conducted experiments pertaining to **Figure 5**. SS wrote and edited the manuscript, with KT finalizing the submission. All authors read and approved the manuscript. All authors contributed to the article and approved the submitted version.

## Funding

This research work is supported by the National University of Singapore, under its Department of Surgery Seed Grant (N-176-000-100-001), and Yong Loo Lin School of Medicine ‘Blastocystis under One Health’ grant (NUHSRO/2022/074/NUSMed/Blastocystis/LOA). SS acknowledges the support of the SINGA PhD Research Scholarship. EK was supported by the Ministry of Education – Post-Doctoral Fellowship from the National University of Singapore. CH was supported by the Ministry of Education research scholarship. LT was supported by the China Scholarship Council (202006330097). JWW would like to acknowledge support by the NUS NanoNASH Program (NUHSRO/2020/002/NanoNash/LOA) and the NUS Yong Loo Lin School of Medicine Nanomedicine Translational Research Program (NUHSRO/2021/034/TRP/09/Nanomedicine).

## Acknowledgments

The authors acknowledge the excellent administrative and technical support of Ms. Geok Choo Ng. The authors also thank the following personnel for their excellent technical assistance and advice: Mr. Cyrill Kafi Salim for his technical advice on EV uptake imaging protocols, Ms. Shu Ying Lee for confocal microscopy and Ms. Xiaoning Wang for flow cytometry at the NUS Medicine Flow Cytometry Unit, and Dr. Qifeng Lin and Mr. Teck Kwang Lim at the SINGMASS unit at the National University of Singapore, Singapore.

## Conflict of interests

The authors declare that the research was conducted in the absence of any commercial or financial relationships that could be construed as a potential conflict of interest.

## Publisher’s note

All claims expressed in this article are solely those of the authors and do not necessarily represent those of their affiliated organizations, or those of the publisher, the editors and the reviewers. Any product that may be evaluated in this article, or claim that may be made by its manufacturer, is not guaranteed or endorsed by the publisher.
